# Suppressing Platinum Electrocatalyst Degradation via a High-Surface-Area Organic Matrix Support

**DOI:** 10.1021/acsomega.1c06028

**Published:** 2022-01-19

**Authors:** Milutin Smiljanić, Marjan Bele, Léonard Jean Moriau, John Fredy Vélez Santa, Svit Menart, Martin Šala, Armin Hrnjić, Primož Jovanovič, Francisco Ruiz-Zepeda, Miran Gaberšček, Nejc Hodnik

**Affiliations:** † Department of Materials Chemistry, National Institute of Chemistry, Hajdrihova 19, 1000 Ljubljana, Slovenia; ‡ Laboratory for Atomic Physics, Institute for Nuclear Sciences Vinča, University of Belgrade, Mike Alasa 12-14, 11001 Belgrade, Serbia; § Jožef Stefan International Postgraduate School, Jamova cesta 39, 1000 Ljubljana, Slovenia; ∥ Materials Physics Center (CSIC-UPV/EHU), Paseo Manuel de Lardizabal 5, Donostia-San Sebastián 20018, Spain; ⊥ Department of Analytical Chemistry, National Institute of Chemistry, Hajdrihova 19, 1000 Ljubljana, Slovenia; # Faculty of Chemistry and Chemical Technology, University of Ljubljana, Večna pot 113, 1000 Ljubljana, Slovenia; ∇ University of Nova Gorica, Vipavska 13, 5000 Nova Gorica, Slovenia

## Abstract

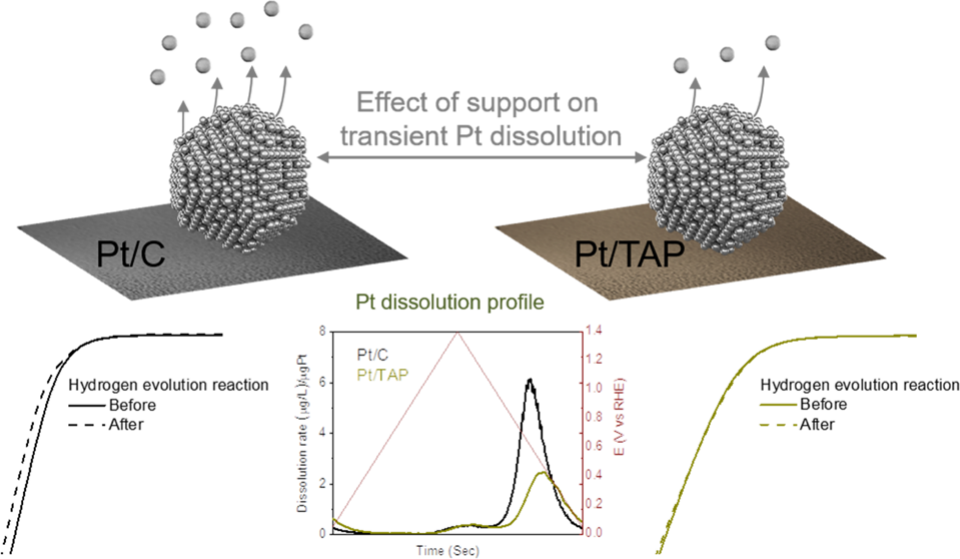

Degradation of carbon-supported Pt nanocatalysts in fuel cells and electrolyzers hinders widespread commercialization of these green technologies. Transition between oxidized and reduced states of Pt during fast potential spikes triggers significant Pt dissolution. Therefore, designing Pt-based catalysts able to withstand such conditions is of critical importance. We report here on a strategy to suppress Pt dissolution by using an organic matrix tris(aza)pentacene (TAP) as an alternative support material for Pt. The major benefit of TAP is its potential-dependent conductivity in aqueous media, which was directly evidenced by electrochemical impedance spectroscopy. At potentials below ∼0.45 V_RHE_, TAP is protonated and its conductivity is improved, which enables supported Pt to run hydrogen reactions. At potentials corresponding to Pt oxidation/reduction (>∼0.45 V_RHE_), TAP is deprotonated and its conductivity is restricted. Tunable conductivity of TAP enhanced the durability of the Pt/TAP with respect to Pt/C when these two materials were subjected to the same degradation protocol (0.1 M HClO_4_ electrolyte, 3000 voltammetric scans, 1 V/s, 0.05–1.4 V_RHE_). The exceptional stability of Pt/TAP composite on a nanoscale level was confirmed by identical location TEM imaging before and after the used degradation protocol. Suppression of transient Pt dissolution from Pt/TAP with respect to the Pt/C benchmark was directly measured in a setup consisting of an electrochemical flow cell connected to inductively coupled plasma-mass spectrometry.

## 1 Introduction

Hydrogen-fed fuel cells are expected to find a broad application in both stationary and mobile devices.^1,2^ Usage of scarce and expensive Pt-based catalysts to run both hydrogen oxidation reaction (HOR) at the anode^3,4^ and oxygen reduction reaction (ORR) at the cathode^5,6^ impedes widespread commercialization of these devices. Another critical issue is the limited lifetime of Pt-catalysts caused by their degradation and subsequent deactivation.^5−8^ Numerous factors influence degradation of Pt-catalysts, such as intrinsic operating conditions inside a fuel cell when the anode is filled with pure hydrogen and the potential at the cathode side is set below 1 V.^9^ However, more significant catalyst deactivation comes from transient conditions during device switching on/off. Namely, shut-down/start-up events lead to uncontrolled conditions in fuel cells, such as air-coexistence with hydrogen in the anode compartment.^10−13^ The presence of air results in unwanted ORR taking place at the Pt anode, which causes corresponding counter reactions at the Pt cathode where local potentials can rise to 1.5–2 V.^12,13^ Consequently, cathode rapidly degrades due to the corrosion of the carbon support. Sudden potential jumps between low and high values on both anode and cathode can also be caused by device switching on/off. These potential spikes are particularly damaging since a major electrochemical Pt dissolution takes place during the transitions between the oxidized and reduced states of Pt surface (and *vice versa*).^14−18^ Dissolved Pt can redeposit back onto existing nanoparticles,^6,19^ or it can precipitate in the fuel cell polymer membrane leading to its failure.^20,21^ In addition, carbon corrosion and coalescence of Pt particles at high potentials contribute to the rapid decay of the electrochemical surface area (ESA) of the Pt catalysts.^6,19^ Similar potential spikes can appear also in water electrolyzers during shut-down/start-up events and affect Pt cathode in the same fashion.

Recently, we have reported on an alternative support material for Pt nanoparticles, an organic matrix tris(aza)pentacene (TAP).^22^ TAP is composed of three branches of tetraazapentacene units and contains a fully aromatic π-conjugated structure. Quinoxaline groups within the organic matrix can undergo reversible reduction, which affects the electrical properties of TAP.^23^ The morphology of TAP offers a large surface area convenient for grafting Pt nanostructures, especially on pyrazine subunits of quinoxaline groups where Pt can be anchored *via* nitrogen complexation.^24,25^ It has been demonstrated that the Pt/TAP composite operates as a selective Pt-anode fuel cell that runs HOR and at the same time effectively blocks ORR.^22^ Such selectivity was attributed to the rich chemistry of the organic matrix that provides a potential dependent conductivity of TAP. In aqueous electrolytes, TAP undergoes reversible hydrogenation at potentials below 0.45 V_RHE_, which enhances its conductivity and enables supported Pt particles to catalyze HOR (and hydrogen evolution). At potentials above this transition, conductivity of TAP is restricted leading to the rather poor ORR activity of Pt/TAP with respect to the Pt/C benchmark. As discussed earlier, such selective catalytic behavior of a fuel cell anode is beneficial for the overall device lifetime.^12,13,22^

Another benefit of the restricted conductivity of TAP at higher potentials could be a suppression of electrochemical oxidation/reduction of supported Pt, which means that aggressive Pt transient dissolution should be limited with respect to state-of-the-art catalysts comprised of Pt nanoparticles dispersed on high-surface-area carbon. Therefore, the Pt/TAP composite is expected to be more resistant to the described potential spikes during transient events in fuel cells. This means that Pt/TAP can provide a multifold benefit for the durability of fuel cells: (i) it protects the cathode catalyst layer by selectively blocking ORR at the anode and (ii) it should act as a stable anode catalyst due to the suppressed Pt dissolution during potential spikes. The first point has been studied in our previous work,^22^ while the second point is yet to be explored, the benefits of which are also relevant for water electrolyzers where Pt cathodes are used to run hydrogen evolution reaction (HER).

In this work, we investigate the electrochemical stability and dissolution of Pt from the Pt/TAP composite and Pt/C benchmark by subjecting them to an accelerated degradation test (ADT) consisting of 3000 voltammetric scans (1 V/s) in a 0.1 M HClO_4_ electrolyte in the potential window between 0.05 V_RHE_ and 1.4 V_RHE_. The purpose of such ADT is to mimic the rapid potential jumps in a fuel cell/electrolyzer during shut-down/start-up events. HER has been chosen as a test reaction to track the impact of the ADT on the activity of Pt/TAP and Pt/C. The ADT results reveal no change in the reactivity of Pt/TAP for the HER before and after degradation, while the apparent decay of HER activity is coupled with a significant drop of the Pt electrochemical surface area of the benchmark Pt/C. Identical location transmission electron microscopy (IL-TEM) is an indispensable tool developed for tracking the degradation of electrocatalytic materials on the nanoscale.^26,27^ Here, Pt/TAP is subjected to IL-TEM analysis before and after the ADT and exceptional stability of the composite is demonstrated. Coupling of the electrochemical flow cell (EFC) with an ICP-MS device (EFC-ICP-MS) is a powerful method for detection and quantification of extremely low amounts of electrochemically dissolved metals.^16,28^ Using the EFC-ICP-MS setup, we have been able to confirm notably lower Pt dissolution from the Pt/TAP composite with respect to the Pt/C analogue. Improved stability of Pt/TAP is a consequence of potential-dependent conductivity of TAP support, which is evidenced directly by *in situ* electrochemical impedance spectroscopy (EIS) measurements.

## 2 Results and Discussion

A Pt/TAP composite with 16 wt % Pt loading has been used in this study, and a full characterization of this material is provided in our previous work.^22^ Briefly, the carbon system in the pristine TAP has a porous hierarchical structure rich with holes and channels, which has a capacity to provide a high surface area for dispersing Pt nanocatalysts. The Pt/TAP composite contains different nanometer-sized Pt structures, including single atoms, nanoparticles with diameters below 5 nm, and nanowires with diameters between 2 and 10 nm and a length up to 200 nm. Such distribution is caused by the differences in the local structure and uniformity of the TAP support, while also the presence of nitrogen in the pyrazine subunits of quinoxaline groups can lead to the preferable Pt anchoring. In the case of the Pt/C benchmark (20 wt %, Premetek), TEM imaging (not shown) revealed well-distributed and uniform-sized Pt nanoparticles with diameters mostly between 1 and 3 nm.

**Figure 1:**
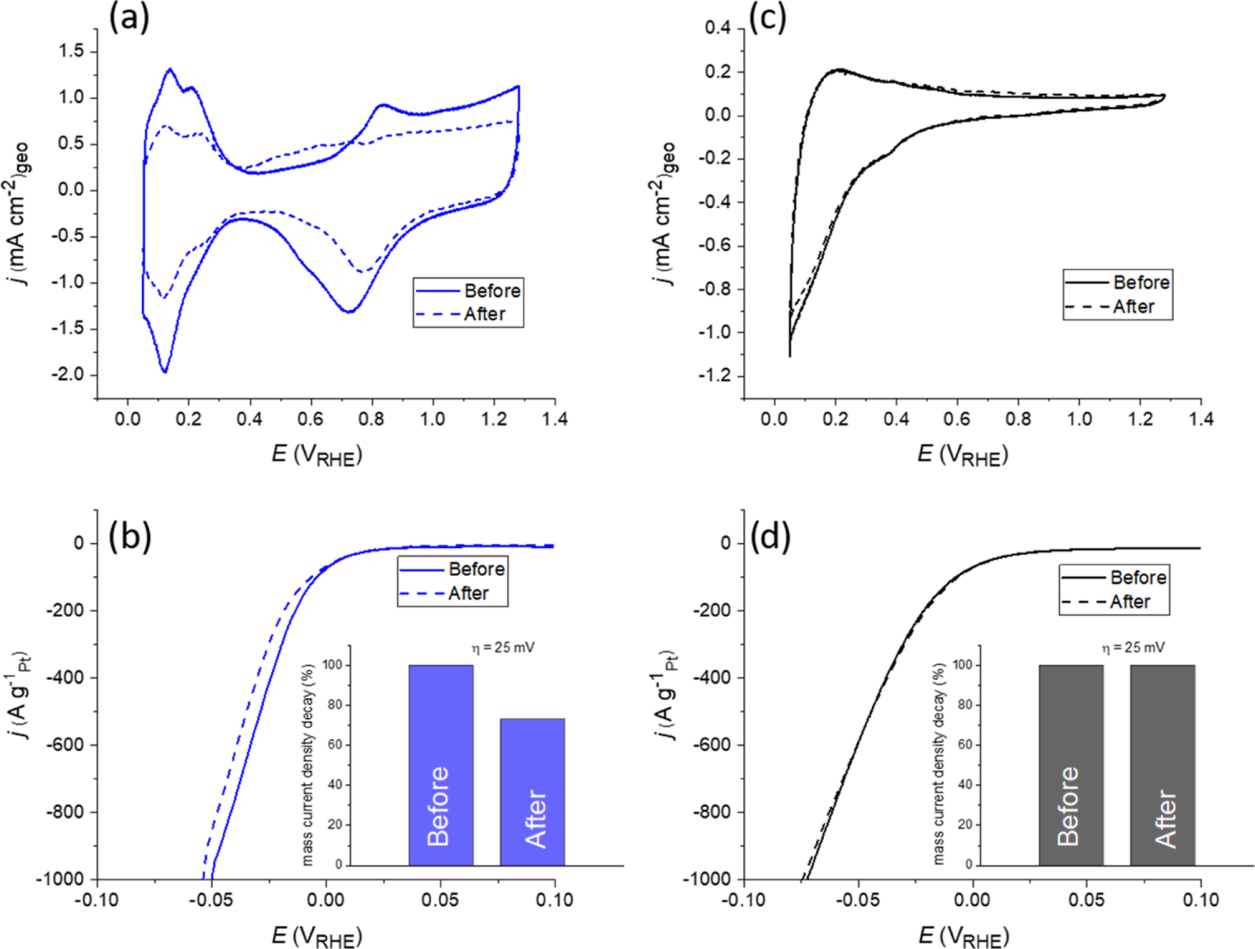
Results of the ADT performed for Pt/C and Pt/TAP catalysts: (a) CVs (50 mV/s) and (b) HER polarization curves (10 mV/s) of Pt/C taken before and after the ADT; (c) CVs (50 mV/s) and (d) HER polarization curves (10 mV/s) of Pt/TAP taken before and after the ADT.

Results of the ADT procedure performed for Pt/C and Pt/TAP are presented in Figure 1. In the case of Pt/C, cyclic voltammograms (CVs) given in Figure 1a show the well-known voltammetric fingerprints of Pt, such as H_upd_ peaks and Pt oxidation–reduction features.^29^ Comparison of CVs collected before and after the ADT shows that Pt/C suffered a significant degradation. ESA of Pt-based catalysts can be extracted by integrating H_upd_ peaks.^29^ A comparison of the charge corresponding to the H_upd_ process on Pt/C before and after ADT reveals a 37.5% loss of Pt ESA. Such Pt/C degradation is accompanied by a decay in the HER activity, Figure 1b. In the case of the novel Pt/TAP composite, CVs given in Figure 1c show only reversible TAP protonation/deprotonation peaks at *E* < 0.45 V_RHE_, which mask H_upd_ peaks on supported Pt. At higher potentials, Pt-oxidation/reduction features are also not visible, which can be explained by the restricted conductivity of the TAP support. Since no Pt-related features are discernible, it is impossible to account for the changes in Pt ESA caused by the ADT. Regarding HER, the activity of Pt/TAP for this reaction remained fully stable at the end of the ADT, as evident from Figure 1d. We should note that Pt/C benchmark is more active than Pt/TAP for HER, and the initial difference in the activities measured at the current density of 10 mA/cm^2^ (normalized to the geometric surface area) equals 20 mV in the favor of Pt/C. This can most likely be ascribed to the difference in Pt ESA of the two samples, as it is reasonable to assume that Pt/C has a larger ESA than Pt/TAP due to the smaller size of Pt particles. Another option is that the carbon support provides a higher conductivity and/or a better mass transport of hydrogen species in/from the catalyst layer with respect to TAP. However, the focus of the present study is on the stable running of HER on Pt/TAP when the catalyst is exposed to rapid potential spikes, which is important for stable operation of the energy conversion devices without power losses and/or fluctuations. Clearly, such stability cannot be provided by Pt/C. For instance, Pt/C mass activity measured at an overpotential of 25 mV dropped down by 27% at the end of the ADT, while for Pt/TAP it remained fully stable. This means that further cycling of both materials will result in the eventual matching of their activities, while at some point Pt/TAP might become even more active than Pt/C. However, we underline that improving the initial HER/HOR activity of the Pt/TAP composite to match the activity of Pt/C will be a matter of further efforts in our group.

Overall, both the voltammetric response and the HER activity of Pt/TAP were unaffected by performed ADT. Since Pt ESA in Pt/TAP cannot be measured, some decay of Pt ESA in Pt/TAP after ADT cannot be excluded. However, we find it reasonable to assume that this decay is less significant than for Pt/C. This assumption will be further supported by IL-TEM, EFC-ICP-MS, and EIS experiments.

Due to the general importance of Pt/C catalysts for fuel cell applications, their degradation has been thoroughly studied by developing advanced methods, such as identical location electron microscopies.^6,19,26,30−33^ In the studies of Mayrhofer^19,26^ and Hodnik,^32,33^ Pt/C benchmark catalysts were subjected to degradation protocols similar to the one used in this work. The significant decay of Pt ESA, comparable to the one observed in Figure 1a, was a consequence of degradation of Pt/C *via* different mechanisms, including dissolution, particle detachment, agglomeration, and Ostwald ripening.

**Figure 2:**
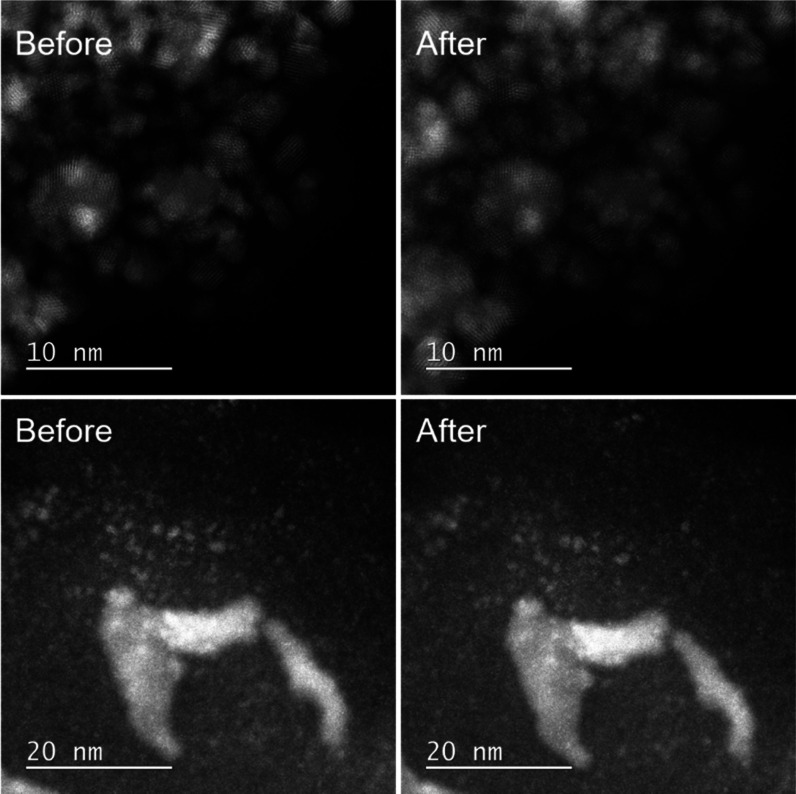
IL-TEM imaging of Pt/TAP before and after ADT. The upper panel images show a region with small Pt nanoparticles, while the lower panel images show a region with larger Pt nanostructures.

Identical location transmission electron microscopy (IL-TEM) is an indispensable tool for direct observation of structural changes of electrocatalytic materials at the nanoscale level.^19,26,27^ A set of representative IL-TEM images of Pt/TAP before and after ADT is reported in Figure 2. Pt/TAP contains different Pt nanostructures, as discussed in detail in our previous work.^22^ Upper panel IL-TEM images show a representative location of Pt/TAP with small Pt nanoparticles, which bear a significant portion of Pt ESA in the Pt/TAP. Despite their rather small size, no notable changes, such as particles missing due to the dissolution or detachment, or particle growth due to the redeposition or agglomeration, can be observed, which points out to the exceptional stability of the Pt/TAP sample. Lower panel images show a typical region with a more robust nanowire-like Pt structure, which also remained rather stable after the ADT. Moreover, it can be seen that the surrounding small-sized Pt nanoparticles did not undergo significant degradation, corroborating the trends from the upper panel images. We should mention that a careful inspection of the IL-TEM images does reveal some minimal changes, such as a few particles missing (visible in the lower panel images). In our opinion, this is expected since some Pt dissolution from Pt/TAP was detected by EFC-ICP-MS (see below). In general, it can be concluded that IL-TEM imaging showed that all Pt nanostructures within Pt/TAP remained stable after the ADT. A comparison with the literature^19,26^ shows that Pt/TAP is more durable than Pt/C when exposed to rapid potential cycling.

**Figure 3:**
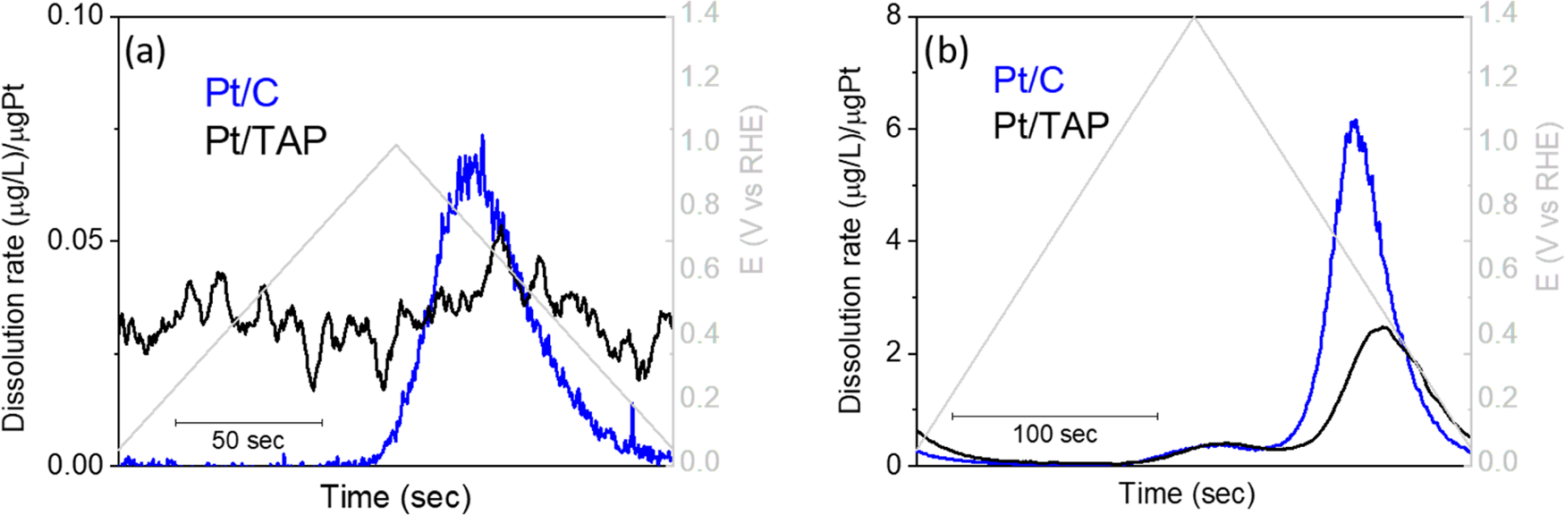
Pt dissolution profiles for Pt/C and Pt/TAP obtained during voltammetric scans at a scan rate of 10 mV/s in the potential range: (a) 0.05–1 V_RHE_ and (b) 0.05–1.4 V_RHE_. The background correction for Pt/TAP could not be properly performed due to low Pt dissolution, which merges with the background signal.

EFC-ICP-MS is another valuable method developed for tracking of online dissolution of metals during relevant electrochemical treatments.^16,28,34^ To compare the extent of Pt dissolution from Pt/C and Pt/TAP, we have subjected these materials in the EFC-ICP-MS setup to slow voltammetric scans (10 mV/s) and monitored Pt dissolution signal in two different potential regions, namely 0.05–1.0 V_RHE_ and 0.05–1.4 V_RHE_. The collected results are presented in Figure 3.

Slow scans in the potential region 0.05–1.0 V_RHE_, Figure 3a, show one peak corresponding to transient Pt dissolution for Pt/C.^15,16^ In the case of Pt/TAP, the Pt dissolution signal deviates from zero because it could not be properly corrected for the background. This is a consequence of particularly low Pt dissolution and the absence of a distinguished transient dissolution peak, which is merged into the background signal and can hardly be recognized as a broad wave in the dissolution profile of Pt/TAP. Clearly, dissolution of Pt from Pt/TAP is far less sensitive to the applied potential sweep and notably suppressed with respect to Pt/C. In the broader potential window of 0.05–1.4 V_RHE_, Figure 3b, Pt dissolution from Pt/C increases due to the higher upper potential limit. The first (lower) peak corresponds to the anodic dissolution, while the second (higher) corresponds to the cathodic transient dissolution during reduction of Pt-oxide.^15,16^ The same peaks appear in the dissolution profile of Pt/TAP, meaning that the conductivity of the TAP support is not fully switched-off and that oxidation/reduction of supported Pt takes place to some extent. Alternatively, Pt dissolution may come from particular interconnected larger Pt structures where restricted conductivity of TAP is less effective. More importantly, the total amount of dissolved Pt during one slow scan from Pt/TAP is two times lower with respect to Pt/C (normalized to the Pt loading).

**Figure 4:**
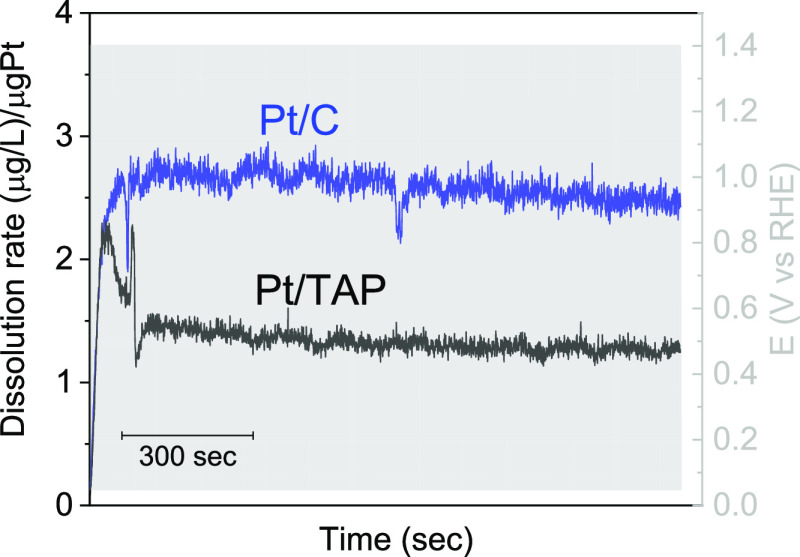
Pt dissolution from Pt/C and Pt/TAP during 500 rapid voltammetric scans (1 V/s) recorded in the potential region between 0.05 V_RHE_ and 1.4 V_RHE_ in a 0.1 M HClO_4_ electrolyte.

Furthermore, we studied dissolution of Pt from Pt/TAP and Pt/C during 500 rapid voltammetric potential cycles used in ADT to simulate start–stop conditions in fuel cells/electrolyzers, Figure 4. In accordance with dissolution during slow scans, the amount of dissolved Pt from Pt/C during rapid voltammetric scans is ∼2 times higher than from Pt/TAP. Overall, the lower Pt dissolution is in agreement with ADT results (Figure 1) and with IL-TEM imaging (Figure 2), which all together point out to the enhanced stability of Pt/TAP with respect to Pt/C.

**Figure 5:**
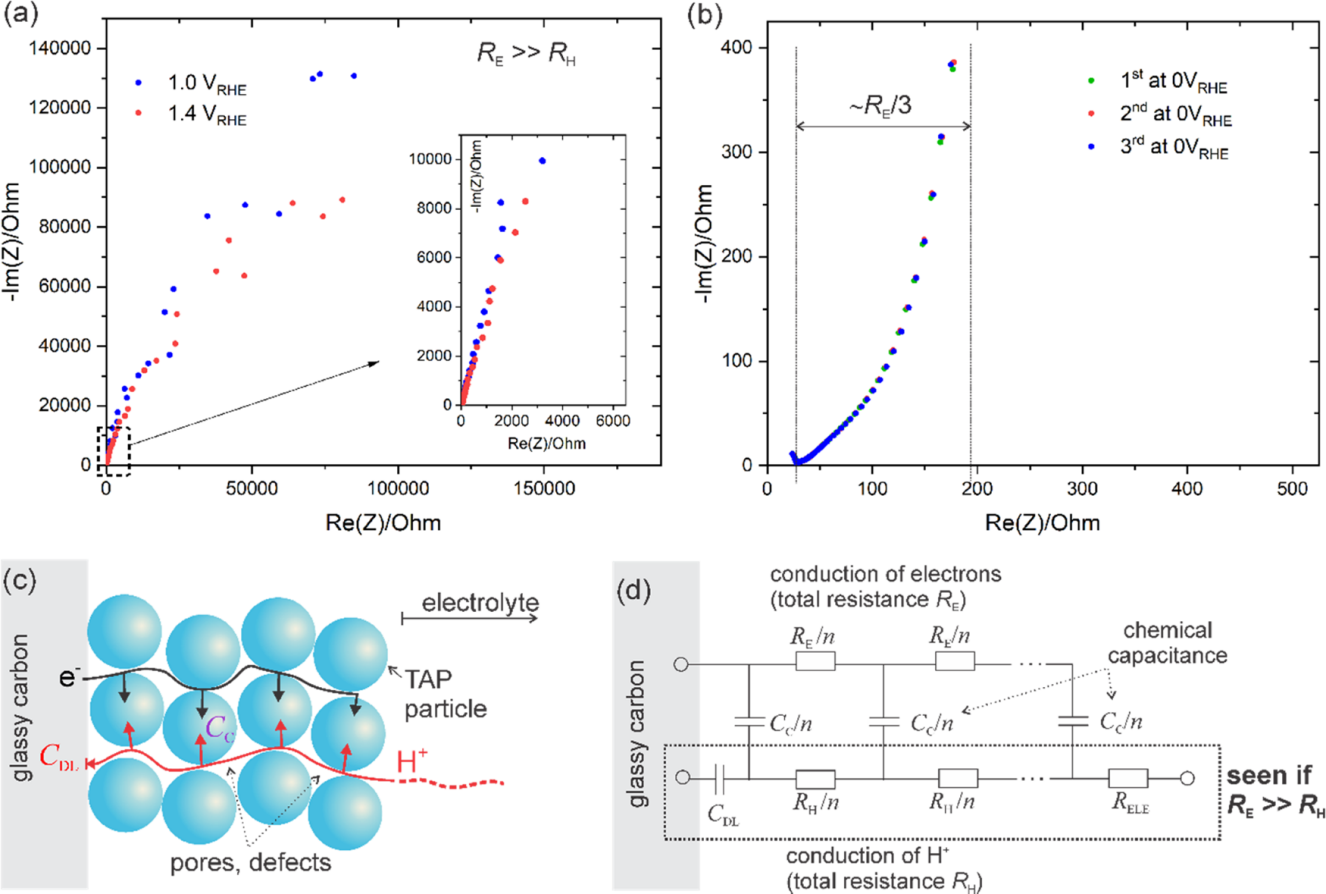
Impedance spectra measured at (a) high and (b) low potentials *vs* RHE. (c) Schematic of ionic and electronic transport and insertion in the defect-rich TAP film deposited on a glassy carbon electrode. (d) Transmission line model corresponding to the transport/insertion mechanism(s) in panel (c). The whole transmission line successfully describes the impedance of the hydrogenated state. The part in the dashed box corresponds to the nonhydrogenated state when insertion of hydrogen/electrons is not possible. *R*_ELE_ corresponds to the resistance of the electrolyte between the working and reference electrodes (in the range of 25 Ω in the present electrode configuration).

We ascribe the enhanced stability of the Pt/TAP composite to the rich chemistry of the TAP support, which provides a tunable conductivity of this organic material in aqueous media. To confirm the increase in electronic conductivity of TAP upon hydrogenation, we performed an *in situ* experiment by measuring EIS at extreme potentials corresponding to protonated and deprotonated states of TAP. The obtained results together with their interpretation are given in Figure 5. Specifically, we can see that at high potentials, i.e., when TAP is deprotonated, the impedance spectrum consists of a slightly tilted vertical line, while the impedance values at low frequencies (<0.1 Hz) typically surpass 100 kΩ (Figure 5a). At low potentials, i.e., the protonated state of TAP, the medium-to-low part of the impedance spectrum consists of a 45° line, which gradually becomes steeper, turning toward a vertical line at the lowest frequencies (Figure 5b). Notably, the impedance values are typically 100–1000 times smaller than at high potentials. Both results can be explained in a straightforward way by referring to established models in the field of insertion battery electrodes. Here, we base our analysis on the general transmission line model^35^ derived from Newman’s theory of porous electrodes.^36^ For the present configuration, the general model is simplified so that only the most important contributions shown schematically in Figure 5c are taken into account. Specifically, at high potentials, no electron/ion insertion is expected so only the movement of hydrogen ions across the film defects occurs. At the surface of glassy carbon, the mobile charges are stored in the corresponding double layer denoted by the double-layer capacitor, *C*_DL_. This situation is described by the very simple transmission line shown in the dashed box in Figure 5d. Conversely, at low potentials, both hydrogen ions, as well as electrons, can be moved and stored in TAP particles. This is described by the whole model shown in Figure 5d. The model reproduces very well both the shape and the size of spectra in Figure 5a,b. From the detailed analysis (not shown) all of the values of elements in the model can be uniquely extracted. Here, we are particularly interested in the value of the electronic resistance, *R*_E_, which roughly corresponds to the triple value of the section shown in Figure 5b. Thus, we estimate that at low potentials (hydrogenated state) the value of the electron resistance is about 500 Ω. This is several orders less than at high potentials where the estimated resistance is >10^5^ Ω (this is the lowest estimation, and the real value might be even much higher). Therefore, EIS clearly demonstrates the difference between the nonhydrogenated and hydrogenated states of TAP. In the former, the electronic resistance is extremely high so no insertion processes are possible (insertion requires a coupled diffusion of both electrons and hydrogen ions). At low potentials, the electron resistance drops to low enough values that insertion of the charge into TAP particles becomes possible.

In addition to tunable conductivity of the TAP support, another possible reason for the lower Pt dissolution from Pt/TAP could be found in the particle size effect,^16^ since larger Pt nanostructures are present in Pt/TAP than in Pt/C. As mentioned earlier, the Pt/C benchmark contains uniform-sized nanoparticles in the range of 1–3 nm in diameter, while Pt/TAP contains single atoms, small Pt nanoparticles with a diameter below 5 nm and also larger nanowires.^22^ Degradation of nonuniform samples usually starts with the dissolution of the smallest particles, as recently shown by our group in the case of Au/C catalyst.^37^ The significant decay of Au ESA during degradation test originated from the dissolution of the smallest particles in the sample (<∼5 nm), which give the highest contribution to the overall ESA. In contrast, larger Au nanoparticles (>∼20 nm) remained practically intact. Reflecting on Pt/TAP, this means that degradation should start with the dissolution of single atoms^38^ and small nanoparticles.^16^ However, we clearly showed by IL-TEM that not only robust nanowires are stable in Pt/TAP but also even very small Pt nanoparticles. EIS measurements confirmed the proposed concept of tunable conductivity of the TAP support. Therefore, we believe that the particle size effect does not have a decisive role on suppressed Pt dissolution from Pt/TAP with respect to Pt/C. In fact, larger Pt nanowires could actually contribute to the observed dissolution, since there is a higher probability that they will form larger interconnected structures where the restricted conductivity of TAP will be less effective.^12,22^

## 3 Conclusions

In this work, we present a new concept for suppressing Pt electrochemical dissolution using the organic matrix tris(aza)pentacene as a support for Pt nanocatalysts. Electrochemical stability and Pt dissolution from the Pt/TAP composite and from the Pt/C benchmark were studied by subjecting these catalysts to the same degradation test (3000 voltammetric scans, 1 V/s, 0.05–1.4 V_RHE_, 0.1 M HClO_4_). Pt/C suffered notable degradation, as revealed by the decay of both the Pt ESA and its catalytic activity for HER, which was used as a test reaction. In contrast, activity of Pt/TAP for HER was not affected by the ADT. IL-TEM imaging revealed exceptional stability of the Pt/TAP composite during the ADT, since no discernible changes in the structure of the sample at the nanoscale level were found. Using the EFC-ICP-MS setup, we have demonstrated that significantly lower Pt dissolution occurs from Pt/TAP than from Pt/C. EIS revealed that the conductivity of TAP is indeed dependent on the potential and that it significantly changes upon protonation/deprotonation of TAP. In particular, the electronic resistance of deprotonated TAP (*E* > 0.45 V_RHE_) is extremely high, which limits the extent of Pt oxidation/reduction, hence transient Pt dissolution is also limited. In contrast, the electronic resistance of TAP drops down for several orders of magnitude upon protonation (*E* < 0.45 V_RHE_), which enables supported Pt particles to run HER/HOR. Such behavior of the Pt/TAP catalyst is beneficial for the durability of energy conversion devices from several points of view: (i) it provides a stable running of hydrogen reactions without power losses or fluctuations; (ii) it increases the lifetime of the polymer membrane by reducing the amount of dissolved Pt that can precipitate on it, and (iii) it provides the fuel cell Pt anode able to selectively run the HOR and to suppress the ORR.

## 4 Experimental Section

Synthesis of the organic matrix tris(aza)-pentacene (TAP) has been conducted according to the previous reports.^22,39^ A mixture of 2,3-diaminophenazine (Fluorochem 95%, 1 g, 4.80 mmol), hexaketocyclohexane octahydrate (Fluorochem 95%, 0.50 g, 1.60 mmol), and deoxygenated acetic acid (Fluorochem, 45 mL) was refluxed for 16 h. The product was cooled down to room temperature followed by filtering and washing with acetone and acetic acid, drying, and further treatment with Soxhlet extraction with ethanol over 12 h. Finally, a black powder was obtained after overnight drying in a vacuum at 80 °C. Synthesis of the 16 wt % Pt/TAP composite is also reported in ref (22). Briefly, a solution of 60 mg of tetraammineplatinum (II) nitrate (Alfa Aesar, product number: 88,969) in 1 mL of water was mixed with 100 mg of TAP powder at 50 °C until evaporation. About 1 mL of ammonia solution 25% (Merck, product number: 105,423) and 1 mL of hydrazine hydrate 50–60% (Sigma-Aldrich, 225,819) were added, and the mixture was treated thermally in a 5% H_2_/Ar gas mixture with a constant flow of 100 cm^3^/min and a pressure of 1 atm, while the temperature was programmed to increase up to 300 °C at a rate of 2 °C/min, followed by cooling down to ambient temperature with a rate of 3 °C/min. The benchmark platinum catalyst used in this work for comparison with Pt/TAP was a commercial Pt/C (20 wt %) catalyst purchased from Premetek.

Evaluation of the stability of Pt/TAP and Pt/C catalysts was performed in a rotating disc electrode setup (RDE). Glassy carbon (GC) working electrodes were cleaned by hand polishing with a 0.05 μM alumina slurry, followed by removal of alumina residues in an ultrasonic bath in Milli-Q water. Catalysts were deposited on GC RDEs in the form of thin films. Catalyst inks were prepared by adding 1 mg of Pt/TAP or Pt/C in 1 mL of Milli-Q water. After mixing, inks were subjected to an ice-cooled ultrasonic bath to obtain fine dispersions. 25 μL of Pt/C or Pt/TAP was drop-casted directly from the ultrasonic bath onto clean and dry GC electrodes and left to dry slowly and in clean conditions. This resulted in Pt loadings of 5 and 4 μg for Pt/C and Pt/TAP samples, respectively. Such a difference should not play a role for this study that focuses on the stability of Pt/TAP. Finally, 5 μL of Nafion solution (5 wt % in a mixture of lower aliphatic alcohols and water, Sigma-Aldrich) diluted in isopropanol (1:50) was added on top of the dried catalyst film to ensure good adhesion.

Electrochemical measurements were performed in a classic three-electrode setup, with a glassy carbon rod as the counter electrode and Ag/AgCl as the reference electrode. Since chloride leakage from reference Ag/AgCl could have a rather significant impact on the Pt dissolution studies, special care was taken to prevent diffusion of chlorides from the reference electrode compartment (REC) to the working electrode compartment (WEC) by separating them with an electrolytic bridge. Such a setup has been proven to effectively prevent diffusion of chlorides from REC to WEC; hence, the possible impact of chlorides on the degradation studies is eliminated.^40^ All potentials are further reported with respect to the reversible hydrogen electrode (RHE). Before and after each experiment, the electrochemical cell with all components was cleaned by boiling in distilled water followed by extensive rinsing with Milli-Q water. All electrochemical measurements were performed in an Ar-saturated 0.1 M HClO_4_ electrolyte prepared by mixing appropriate amounts of perchloric acid (Rotipuran Supra 70%, Carl Roth) and Milli-Q water.

Before stability tests, Pt/C and Pt/TAP catalysts were electrochemically activated to ensure full wetting of the catalyst film and to reach a stable initial voltammetric response. Pt/C activation consisted of 200 voltammetric cycles at 300 mV/s in the potential window of 0.05–1.2 V_RHE_, while for Pt/TAP, 50 scans were performed at 200 mV/s in the potential range of 0.05–1.28 V_RHE_. After activation, cyclic voltammograms at 50 mV/s in the potential range of 0.05–1.28 V_RHE_ were recorded to obtain the initial state of the catalysts before ADT. The hydrogen evolution reaction was chosen as the test reaction to observe the impact of the degradation on the electrocatalytic activity of Pt/C and Pt/TAP. HER polarization curves were recorded in the same deaerated 0.1 M HClO_4_ electrolyte starting from 0.2 V_RHE_ and going down to −0.1 V_RHE_ at a scan rate of 10 mV/s.

Stability testing of Pt/C and Pt/TAP materials was performed by an accelerated degradation test consisting of 3000 rapid voltammetric cycles at a scan rate of 1 V/s in the potential window of 0.05–1.4 V_RHE_. At the end of the ADT, CV and the HER polarization curves for both catalysts were recorded (as described above) for comparison with their initial state. All electrochemical measurements were performed on a BioLogic SP-300 potentiostat with iR compensation.

Electrochemical impedance spectroscopy (EIS) measurements have been conducted to investigate potential-dependent changes in the conductivity of the TAP support. For that purpose, a film of TAP on the GC electrode was prepared in the same way as for Pt/C and Pt/TAP. EIS spectra were measured at different potentials corresponding to protonated and deprotonated states of the TAP support. The used frequency range was between 50 mHz and 100 kHz, while an amplitude of 10 mV was applied.

To observe degradation of the Pt/TAP material during ADT on a nanoscale level, identical location TEM imaging was applied. For that purpose, a gold TEM finder grid was coated with the Pt/TAP catalyst and subjected to TEM imaging in the pristine state. Afterward, the grid was placed into a modified floating electrode setup, described in detail in our previous work.^41^ This setup enables performing electrochemistry directly on a Pt/TAP-coated TEM grid as the working electrode. The same protocol as in RDE was used for degradation of Pt/TAP. The grid was then dried, and TEM imaging was performed on identical locations to provide a direct comparison with pristine sample. Scanning transmission electron microscopy imaging was performed with a JEOL ARM200CF operated at 80 kV.

The electrochemical flow cell (EFC) connected with an ICP-MS device (EFC-ICP-MS) was used for the potential resolved analysis of the dissolved platinum from Pt/C and Pt/TAP catalysts. The EFC-ICP-MS system is described in detail in our previous studies.^34,37,42,43^ Briefly, the setup consists of an EFC connected to an ICP-MS instrument (Agilent 7900, Agilent Technologies), equipped with a MicroMist glass concentric nebulizer and a Peltier cooled Scott-type double-pass quartz spray chamber. The EFC is custom-made from polyether ether ketone (PEEK) based on a design of a commercial cell (crossflow cell, BASi), where two glassy carbon disks (3 mm in diameter) serve as the working and counter electrodes. GC disks were cleaned by polishing with a 0.05 μm Al_2_O_3_ paste and then removal of alumina residues was ensured by exposing them to an ultrasonic bath in Milli-Q water. The catalyst ink was drop cast (5 μL) on one of the GC disks and left to dry under ambient conditions. Dried films were covered with a drop of the Nafion–isopropanol mixture. The electrolyte (0.1 M HClO_4_) flowed in the direction from the counter electrode to the working electrode at a constant flow of 400 μL/min with a mechanical syringe pump. An Ag/AgCl reference electrode with a ceramic frit (MW-2030, BASi) was used as a reference electrode. The standardization curve was determined based on the standard solutions containing 1, 2, 5, 10, 20, 50, and 100 ppb of Pt.
